# Clinical Case of a 20-Year-Old Man With Myeloneuropathy Due to Vitamin B12 Deficiency From Nitrous Oxide Abuse: A Case Report

**DOI:** 10.7759/cureus.86225

**Published:** 2025-06-17

**Authors:** Petar Vasilev, Ekaterina Viteva, Georgi S Slavov

**Affiliations:** 1 Department of Neurology, Medical University of Plovdiv, Plovdiv, BGR

**Keywords:** myeloneuropathy, myelopathy, nitrous oxide, subacute combined degeneration, vitamin b12 deficiency

## Abstract

Vitamin B12 deficiency-associated myeloneuropathy is a combination of subacute combined degeneration of the spinal cord and peripheral neuropathy. There are various causes of vitamin B12 deficiency, including diet mistakes, gastrointestinal diseases, genetic disorders, and medication intake.

We present a clinical case of a 20-year-old male with myeloneuropathy due to vitamin B12 deficiency. The symptoms had been developing for four to five months, and upon admission to our clinic, the patient was presented with superficial and deep sensation deficit, distal paresthesia, muscle weakness, spastic-paretic gait, and the feeling of incomplete emptying of the bladder after urinating. The MRI revealed hyperintense lesions in cervical and thoracic areas of the spinal cord, and the electroneurogram indicated decreased conduction speed and severe axonal damage of the peripheral nerves. The patient admitted about transitional abdominal pain for years and chronic overuse of nitrous oxide (*laughing gas*) before symptom onset. Treatment with intramuscular vitamin B12 was started. The patient was also diagnosed with chronic gastritis and treated with antibiotics for 10 days and a proton pump inhibitor for 30 days. After a nine-month treatment course with vitamin B12, the patient significantly improved, although some mild symptoms persisted.

The clinical case demonstrates the importance of taking into consideration vitamin B12 deficiency when diagnosing myelopathy and/or peripheral neuropathy. Vitamin B12 deficiency-associated neurological conditions are easy to treat and potentially fully reversible if diagnosed on time.

## Introduction

Vitamin B12, commonly referred to as cobalamin, is typically sourced from animal-based foods and plays a crucial role in the production of deoxyribonucleic acid (DNA), fatty acids, and myelin [1]. Its deficiency may cause megaloblastic anemia and various potentially reversible neurologic conditions such as myelopathy, peripheral neuropathy, dementia, and optic neuropathy.

Nitrous oxide is а colorless, tasteless, and odorless gas that induces relaxation and euphoria. Based on the global drug survey conducted in 2018, nitrous oxide holds the position of the seventh most common recreational substance globally, not counting nicotine, caffeine, and alcohol [2,3]. We describe a case of myeloneuropathy in a young male as a result of B12 deficiency due to nitrous oxide abuse.

## Case presentation

**Figure 1:**
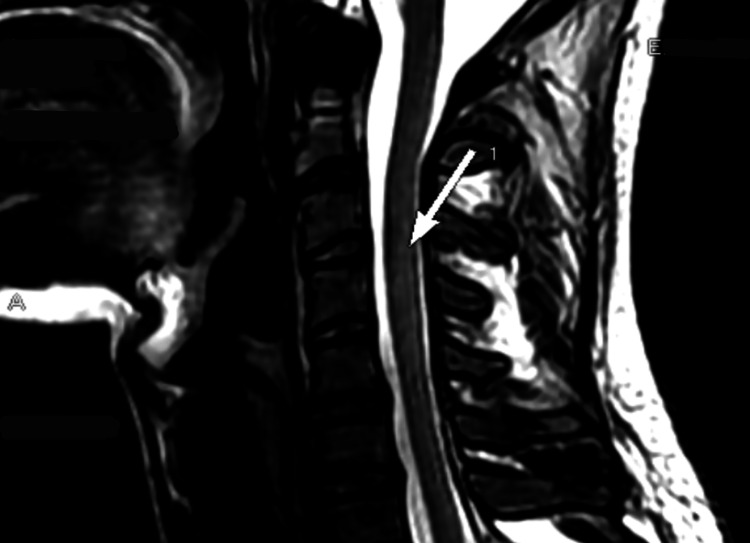
T2-weighted sagittal MRI of the cervical spine revealing hyperintense lesions in the dorsal columns of the spinal cord.

**Figure 2:**
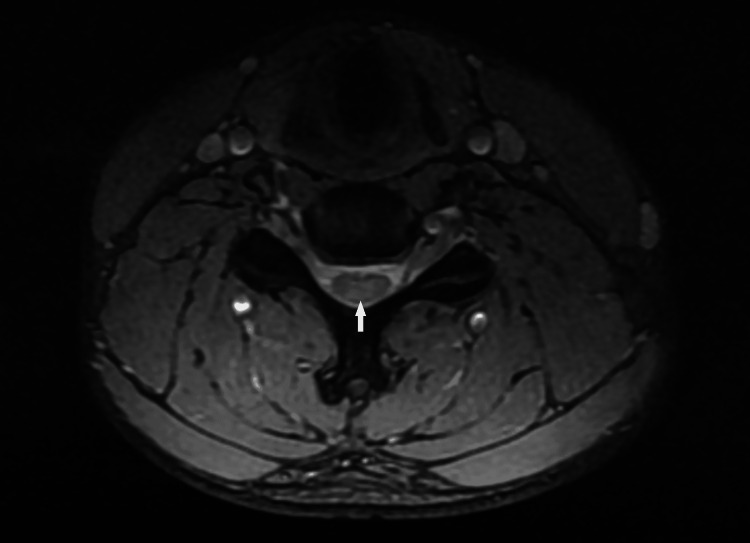
T2-weighted spoiled gradient echo (MERGE) transverse MRI of the cervical spine revealing hyperintense lesions in the dorsal columns of the spinal cord.

We present a clinical case of a 20-year-old, physically active patient with subacute combined degeneration (SCD) of the spinal cord and peripheral neuropathy as a result of vitamin B12 deficiency. The symptoms began four to five months before hospitalization, with transient numbness and tingling in the fingers on both arms. Several days before visiting the neurologist, the patient developed paraparesis in the legs and could hardly walk. Serum levels of vitamin B12 were below the reference range. The MRI revealed poorly defined hyperintense foci on T2-weighted and fluid-attenuated inversion recovery (FLAIR) images along the spinal cord, which appeared in the axial view as symmetrical lenticular areas forming an *inverted V* pattern (Figures 1, 2). The lesions were localized in the cervical and thoracic medulla and did not change their characteristics after contrast application. The patient started taking vitamin B12 orally and was hospitalized 10 days later for further evaluation.

Upon admission, the patient presented with a spastic-paretic gait, distal hypesthesia from T6-T7 (more pronounced in the right foot), decreased tendon reflexes in the upper extremities, and absent reflexes in the lower extremities. Plantar reflexes were absent. Examination revealed impaired graphesthesia in the lower extremities, disturbed joint position sense in the right leg, a positive Romberg sign, and dynamic ataxia in the right leg during the heel-to-shin test. The patient also reported having experienced periodic abdominal pain for several years and, more recently, frequently inhaling balloons filled with *laughing gas* (N₂O, nitrous oxide).

Blood tests did not show any hematological changes or inflammation. Following blood serum analysis and cerebrospinal fluid (CSF) collection via lumbar puncture, infectious and autoimmune diseases were ruled out at that time. At the time of hospitalization, serum levels of vitamin B12, folic acid, and methylmalonic acid (MMA) were within normal ranges; however, homocysteine levels were elevated (20.9 µmol/L; reference value <12 µmol/L). It should be noted that these tests were performed after the patient had already been taking oral vitamin B12 for approximately 10 days. Stimulus electroneurography of the fibular and tibial nerves showed absent distal conduction, significantly reduced conduction velocity, and severe axonal damage. Fibrogastroscopy revealed chronic atrophic gastritis, positive for Helicobacter pylori, with moderate chronic inflammation and mild atrophy of the antral mucosa. 

Parenteral application of vitamin B12 was started. The dosing regimen was 1,000 mcg intramuscularly every day for seven days, followed by 1,000 mcg once a week for one month and 1,000 mcg per month for nine months. Treatment for the chronic gastritis was also prescribed: amoxicillin 2,000 mg daily for 10 days, clarithromycin 1,000 mg daily for 10 days, and esomeprazole 40 mg daily for 30 days.

During a nine-month follow-up, the patient gradually improved, and the serum level of vitamin B12 remained within normal range values. However, some symptoms persisted - namely, decreased tendon reflexes in the upper extremities and absence of reflexes in the lower extremities, paresthesia on the plantar surfaces of the feet, impaired graphesthesia in the lower extremities, a positive Romberg sign, and difficulty with dorsiflexion of the feet. At that time, another contrast MRI was performed, which did not reveal any pathological changes in the brain; however, discrete, not well-defined hyperintense inhomogeneity persisted in the myelon, without additional changes.

## Discussion

Vitamin B12 deficiency could lead to various neurological conditions such as myelopathy, peripheral neuropathy, and cognitive deficit. The myelopathy, known as SCD of the spinal cord, is characterized by demyelination-induced degeneration of the dorsal and lateral columns. Peripheral neuropathy could appear separately or superimposed on the SCD, as it is the clinical manifestation in the reported case [4].

We suggest that in our patient, the combination of underlying chronic gastritis and chronic abuse of nitrous oxide led to vitamin B12 deficiency associated with myeloneuropathy. Patients suffering from chronic gastritis produce less hydrochloric acid and/or intrinsic factor. Gastric acid is required to separate cobalamin from the food and saliva proteins, while vitamin B12 absorption in the terminal ileum depends on intrinsic factor, a glycoprotein produced by parietal cells in the stomach [1,5,6].

On the other hand, nitrous oxide alters cobalt in vitamin B12 from a monovalent to a bivalent state, turning it into a biologically inactive metabolite. The correlation between nitrous oxide and vitamin B12 was initially assessed by Henderson, Patt, and Banks in 1968 and later examined by Nunn in 1987 [7].

The laboratory tests did not show megaloblast anemia, increased MCV, leukopenia, and thrombocytopenia, but it must be taken into consideration that only around 30% of patients with neurological conditions have hematological changes [8]. It has been suggested that cellular-level alterations may cause a functional B12 deficit to appear at any serum level [9,10,11]. MMA and homocysteine levels provide greater insights into B12 metabolism. They serve as cobalamin metabolic intermediates, and a B12 deficit is confirmed by their rise. MMA is solely raised in B12 deficiency, although homocysteine can be elevated in both folate and B12 deficiency [12]. In 2020, a meta-analysis was published that indicated that around 30% of patients with SCD have normal or elevated levels of serum vitamin B12 [13].

The differential diagnoses include other demyelinating diseases, with the most pronounced examples being multiple sclerosis and Devic disease (neuromyelitis optica). For prompt treatment, it is essential to confirm or rule out potential alternative diagnoses such as infectious myelopathy and transverse myelitis. Although transverse myelitis has some important features that make it less likely in this situation, it may present similarly to SCD. Demyelination in transverse myelitis does not predominantly affect the dorsal columns and is frequently restricted to one or two spinal levels. This diagnosis is less plausible in our instance because the MRI showed demyelination of the cervical and thoracic spinal cord. In this presentation, infectious myelopathy, such as acquired immunodeficiency syndrome (AIDS)-associated vacuolar myelopathy or HIV, should also be taken into consideration [14]. This diagnosis is even less plausible because our patient has no history of HIV, opportunistic infections, or cancer.

Treatment of vitamin B12 deficiency myeloneuropathy involves managing the underlying cause of the deficiency and providing adequate parenteral (intramuscular) supplementation. There are different dosing regimens for intramuscular treatment depending on the severity of the symptoms. Treatment usually begins with 1,000 mcg of vitamin B12 administered every other day for two weeks, followed by once weekly for three to four weeks, and then once every one to three months [8,14,15]. The intensity of the treatment may differ according to the individual situation.

Prognosis depends on the severity of the initial involvement, with 86% of patients showing partial improvement and only 14% achieving complete neurological recovery [5,16].

## Conclusions

This case is of clinical interest due to the severity of the neurological symptoms and the significant improvement observed during treatment, even though complete regression was not achieved.

Neurological conditions resulting from nitrous oxide overuse are becoming increasingly common among the young population and may lead to permanent disability. Fortunately, if diagnosed early and parenteral treatment is started on time, neurological deficit is potentially fully reversible. The serious repercussions of this issue should be more thoroughly examined, along with an assessment of the ongoing legal status of the substance. If the recreational use of nitrous oxide continues to rise, it will be essential to develop standardized treatment protocols for SCD to ensure consistency in therapy and potentially improve outcomes.
